# Risk formulation, lived experience, and patient leadership in European CAMHS

**DOI:** 10.3389/phrs.2026.1609723

**Published:** 2026-05-04

**Authors:** Nadia Ahmed, Sophie Mae Harrington, Aikaterina Sidiroglou, Paul Louis Fiedler, Hannah Brunskill, Jennifer Hall, Ledia Lazeri, Joao Breda

**Affiliations:** 1 World Health Organization Office on Quality of Care and Patient Safety, World Health Organization, Athens, Greece; 2 World Health Organization Regional Office for Europe, Copenhagen, Denmark

**Keywords:** child and adolescent, leadership, mental health, quality of care, risk assessment

## Introduction

Child and Adolescent Mental Health Services (CAMHS) across Europe face rising demand, uneven provision, and increasing scrutiny regarding safety and quality. One in seven young people in the WHO European Region lives with a mental health condition, yet many systems lack the infrastructure and policy required to respond effectively [1, 2]. Ensuring safety is therefore central to high-quality CAMHS. However, the way services conceptualise and manage risk remains contested.

Historically, risk management has relied on predictive assessment and stratification, categorising young people as low, medium, or high risk. Such approaches lack reliable predictive validity [3, 4], and their institutional authority sits uneasily alongside commitments to meaningful participation, which range from consultation to youth leadership with shared decision-making authority [5]. When risk procedures marginalise young people’s involvement, tensions emerge between protection and agency.

Against this backdrop, how risk is defined and enacted becomes a question of ethics as much as safety. This commentary argues that risk formulation, grounded in lived experience and strengthened through youth and patient leadership, offers a clinically robust and ethically coherent alternative. Lived experience is positioned as knowledge that reshapes how risk is understood, taught, and governed across systems.

## What is risk formulation

Risk stratification reduces complex relational experience to categorical judgement. A young person becomes “high” or “low” risk according to static markers, despite limited evidence that such classifications meaningfully predict future behaviour [4, 6]. These categories do not merely describe risk; they shape response by restricting agency or access to care. Clinical attention narrows, developmental and relational context recedes, and procedural certainty substitutes for shared understanding.

Beyond limited predictive validity, stratified approaches can generate false reassurance, defensive practice, and erosion of therapeutic trust [7]. In England, NICE NG225 (2022) marks a significant policy shift, advising against the use of risk scales to determine treatment pathways and instead emphasizing comprehensive psychosocial assessment grounded in collaboration and context.

Lived experience accounts illuminate the consequences of reductionism. Being labelled “dangerous” without explanation can lead to restriction, surveillance, and exclusion from ordinary developmental activity. What was intended as protection may be experienced as containment. Service-user research similarly identifies “hidden risks” generated by services themselves, including powerlessness, hopelessness, and loss of agency [8]. Labels often travel across services and time, reinforcing stigma and constraining recovery.

Risk formulation offers a substantive alternative. Rather than predicting harm, formulation seeks to understand how risk emerges for a specific individual within a specific context. It integrates vulnerabilities, precipitating stressors, relational dynamics, and protective resources into a shared explanatory account that informs collaborative care planning. NICE NG225 (2022) reinforces this orientation by prioritising psychosocial assessment and therapeutic engagement irrespective of perceived risk level. Risk is reframed as dynamic and contextual rather than inherent; safety becomes negotiated rather than imposed.

## Youth as sites of knowledge

Experiential knowledge as equal in value to clinical and academic expertise [9]. Within risk practice, this principle reframes young people as contributors to understanding how risk is constructed and lived. is.

Narrative is foundational to formulation. Conventional risk practices often abstract behaviour from context, positioning service users primarily through dangerousness [10]. Formulation restores context by situating behaviour within relationships, developmental trajectories, and lived experience. While checklists record incidents as static indicators, narratives reveal antecedents such as shame, relational rupture, fear of consequences, and overlooked protective factors, including trusted relationships and coping strategies. These dimensions, often absent from stratified tools, are central to prevention.

Further, young people report greater openness when invited to explain rather than defend. The shift from confirming behaviour to exploring meaning alters disclosure and strengthens therapeutic alliance [11]. National Institute for Health and Care Excellence [12] aligns with this approach, advising against categorical prediction in favour of collaborative assessment grounded in context and relationship. Youth leadership advances this orientation by promoting shared decision-making and meaningful collaborative safety planning. Co-developed risk formulation exemplifies this participatory practice, integrating contextual factors into a shared account of risk and prevention [6]. Collaborative formulation can support shifts from disempowerment towards agency, enabling young people to reinterpret experience in personally meaningful ways.

## Co-producing training and service change

National Institute for Health and Care Excellence [12] recommends that training in self-harm and risk assessment be co-developed and co-delivered with people who have lived experience, recognising that relational competence cannot be achieved through procedural compliance alone. Co-production models increasingly position lived experience contributors as partners in curriculum design, documentation revision, supervision, and case review.

Within CAMHS, including work undertaken at North East London NHS Foundation Trust by one of the authors, lived experience contributors supported the redesign of risk documentation to replace binary categorisation with dialogic prompts. Training centred lived narratives, encouraged reflection on power and uncertainty, and emphasised shared decision-making. Such initiatives shift education from accreditation towards relational skill-building, aligning with evidence linking therapeutic alliance and open communication to effective risk management [11, 13].

Training alone, however, does not transform culture. Many services remain oriented toward risk aversion and measurable compliance, allowing formulation to be absorbed into bureaucratic routine. Lived experience contributors frequently identify this disjunction between collaborative language and prescriptive practice. Patient leadership intervenes by advocating for positive risk-taking, transparent and reviewable restrictions, and safety plans focused on context rather than identity. They also highlight the necessity of organisational readiness. WHO underscores the need for formalised roles, reflective supervision, and structural support to ensure participation is meaningful rather than symbolic [9].

## Fostering relational safety

Youth and patient leadership plays a critical role in challenging risk-averse cultures in which fear of adverse outcomes shapes decision-making. Procedural compliance and institutional protection can eclipse relational practice [4]. Young people with lived experience illuminate how containment and surveillance may erode trust. Further, restrictive practices justified as protective may paradoxically increase harm by undermining agency and engagement [8]. Trust and open communication are central to effective risk management and suicide prevention [11, 13]. Cultures driven by blame may suppress disclosure, whereas relational safety encourages honesty.

Youth leadership reframes safety as a partnership rather than exclusion. Recovery-oriented frameworks position individuals as experts in their own experiences [14]. Relational practice requires organisational conditions that tolerate uncertainty rather than demand false certainty. Non-punitive cultures and reflective supervision enable collaborative risk management. Through this lens, formulation functions not only as a clinical approach but as a mechanism for cultural transformation.

## Conclusion and implications

The movement from risk stratification to risk formulation aligns directly with WHO’s commitment to participation, rights-based care, and quality improvement [2, 15]. Formulation operationalises participation at the point of care by embedding narrative and shared decision-making within everyday practice. Youth and patient leadership extend this participation into training, governance, and system design.

Embedding lived-experience-led formulation across the WHO European Region signals more than methodological reform. It represents a shift in epistemology and professional responsibility. Risk is understood as relational and dynamic; safety emerges through trust, transparency, and shared responsibility. Transforming risk practice therefore requires cultivating organisational conditions in which relational care can flourish. When grounded in lived experience, risk formulation becomes both clinical evolution and ethical commitment. Services become safer not through predictive certainty, but through collaborative understanding and humane responses.
